# Methane positive small intestinal bacterial overgrowth in inflammatory bowel disease and irritable bowel syndrome: A systematic review and meta-analysis

**DOI:** 10.1080/19490976.2021.1933313

**Published:** 2021-06-30

**Authors:** Arjun Gandhi, Ayesha Shah, Michael P. Jones, Natasha Koloski, Nicholas J. Talley, Mark Morrison, Gerald Holtmann

**Affiliations:** a Faculty of Medicine, The University of Queensland, Queensland, Australia; b Department of Gastroenterology & Hepatology, Princess Alexandra Hospital, Brisbane, Queensland, Australia; c Translational Research Institute, Brisbane, Queensland, Australia; d Department of Psychology, Macquarie University, Sydney, New South Wales, Australia; e Faculty of Health and Medicine, The University of Newcastle, Newcastle, Australia; f University of Queensland, Diamantina Institute, Brisbane, Queensland, Australia

**Keywords:** Irritable bowel syndrome, inflammatory bowel disease, methane, small intestinal bacterial overgrowth, breath tests

## Abstract

Several studies reported a potential role of methane producing archaea in the pathophysiology of irritable bowel syndrome (IBS) and inflammatory bowel disease (IBD). We conducted a systematic review and meta-analysis to assess the prevalence of methane positive small intestinal bacterial overgrowth (SIBO) in IBS and IBD compared with controls. MEDLINE (PubMed) and Embase electronic databases were searched from inception until March 2021 for case-control and prevalence studies reporting SIBO in IBS and IBD. We extracted data from published studies and calculated pooled prevalence of SIBO in IBS or IBD, odds ratios (OR), and 95% CIs, utilizing a random effects model. The final dataset included 17 independent studies assessing the prevalence of methane positive SIBO in 1,653 IBS-patients and 713 controls, and 7 studies assessing the prevalence of methane positive SIBO in 626 IBD-patients and 497 controls, all utilizing breath test for SIBO diagnosis. Prevalence of methane positive SIBO in IBS and IBD was 25.0% (95% CI 18.8–32.4) and 5.6% (95% CI 2.6–11.8), respectively. Methane positive SIBO in IBS was not increased compared to controls (OR = 1.2, 95% CI 0.8–1.7, *P* = .37) but was significantly more prevalent in IBS-C as compared to IBS-D (OR = 3.1, 95% CI 1.7–5.6, *P* = .0001). The prevalence of methane-positive SIBO in patients with IBD was 3-fold lower at 7.4% (95% CI 5.4–9.8) compared to 23.5% (95% CI 19.8–27.5) in controls. The prevalence of methane positive SIBO was significantly lower in Crohn’s disease as compared to ulcerative colitis, (5.3%, 95% CI 3.0–8.5 vs. 20.2%, 95% CI 12.8–29.4). This systematic review and meta-analysis suggests methane positivity on breath testing is positively associated with IBS-C and inversely with IBD. However, the quality of evidence is low largely due to clinical heterogeneity of the studies. Thus, causality is uncertain and further studies are required.

## Introduction

There is emerging evidence that microbial dysbiosis, defined as the alterations in the composition, density and function of the intestinal microbes plays an important role in a variety of gastrointestinal and extraintestinal conditions.^1^^, 2^ Small intestinal bacterial overgrowth (SIBO), a condition where the overall homeostasis of the small intestine becomes dysregulated through presence of altered number and type of microbes is an example of gut microbial dysbiosis.^3^ The current gold standard for diagnosing SIBO remains small bowel aspirate and culture, however in clinical practice breath testing has largely replaced culture methods given the simplicity and noninvasive nature of these tests.^3^ Breath tests are based on the principle that human cells do not produce hydrogen and/or methane gas^4^ and presence of these gases in the human breath indicates the metabolism of (non-digested) carbohydrates by gut microbes.^5^

Although certain *Clostridium* and *Bacteroides* spp. have been proposed to produce methane^6^ microbes from the third Domain of Life – the Archaea – are now widely believed to exclusively fill this metabolic niche. In humans, *Methanobrevibacter smithii*^7^ is the numerically predominant taxon, and principally rely on the production of methane from hydrogen (H_2_) and carbon dioxide (CO_2_) for their sole source of energy.^8^ Methane generally has not been found to have a physiologic role in humans,^9^ and is mainly excreted in flatus (80%), while a certain amount is excreted in breath (20%).^8^ Thus, methane can be detected during breath tests.

More than one third of healthy adult subjects are predominantly methane producers^10^ and there has been increasing interest in the association between methane and constipation^11^ and the potential effect on gastrointestinal intestinal transit.^11,12^ The role(s) of methane and methanogens in chronic diarrheal states such as inflammatory bowel disease (IBD) and pneumatosis cystoides,^13^ has been associated with significantly reduced concentrations of breath methane, as well as reduced methanogen positivity and counts in stool samples. Emphasizing the potential importance of methane production by archaea, the recent guideline of the American College of Gastroenterology on SIBO coined the term “intestinal methanogen overgrowth” (IMO), for methanogens rather than SIBO driven solely by bacteria.^14^ While previous systematic reviews and meta-analysis have assessed the link between SIBO and irritable bowel syndrome (IBS)^15^ or IBD,^16^ there are no systematic reviews, which have specifically explored the role of methane positive SIBO in relation to these conditions.

Hence, we decided to conduct a systematic review and meta-analysis to (1) assess and compare the prevalence of methane positive SIBO in patients with IBS and IBD (and their subtypes) and healthy controls; (2) explore the link between diagnostic modality (type of breath test) and variations in methane SIBO prevalence in patients with IBS and IBD; (3) assess the association between proton pump inhibitor (PPI) use and methane positivity on breath test; (4) assess the link between transit time and methane positivity on breath tests and; (5) assess the effect of antibiotic therapy on symptom improvement in patients with methane positive SIBO.

## Materials and methods

### Search strategy

A comprehensive literature search was performed using MEDLINE(PubMed) and Embase electronic databases from initiation (1966) up to March 2021 for all studies assessing the prevalence of SIBO in patients with IBD, IBS, and/or functional gastrointestinal disorders (FGIDs). The initial search was not limited to specific languages. A further advanced search was conducted. Grey literature was searched with Google and Google Scholar, and the ‘Snowball” method was also utilized which included pursuing through reference lists of articles as well as electronic citations, to identify all relevant articles. Search terms included “methane” OR “CH_4_” OR “breath test” OR “breath analysis” OR “methane breath test” OR “glucose breath test (GBT)” OR “glucose hydrogen breath test” OR “GBT” OR “lactulose breath test (LBT)” OR “lactulose hydrogen breath test (LHBT)” OR “LBT’ OR “LHBT” AND “constipation” OR “transit” OR “motility” OR “irritable bowel syndrome” OR “IBS” OR “irritable colon” OR “colonic inertia” OR “SIBO” OR “SBBO” OR “small bowel bacterial overgrowth (SBBO)” OR “small intestinal bacterial overgrowth” AND “Inflammatory bowel disease” OR “IBD”. Expert assistance was sought from the hospital librarian who helped conduct a detailed literature search strategy which is outlined in in the PRISMA flow diagram.

### Selection of studies

An initial screen of abstracts and titles were conducted independently by two authors (A.G and A.S). Abstracts were eliminated in this initial screening if they were case series, case reports, animal studies; or if they did not investigate the association between methane positive SIBO and IBD or ulcerative colitis (UC) or Crohn’s disease (CD) or the association between methane positive SIBO and IBS. Full texts of the remaining articles were retrieved and reviewed. Studies recruiting unselected subjects meeting diagnostic criteria for IBS and IBD, that reported the prevalence of methane positive SIBO using clinically validated methods,^17^ and compared the prevalence of methane positive SIBO in IBS and IBD patients versus controls were eligible for inclusion. We also included studies that reported the efficacy data after antibiotic treatment of SIBO in IBS and IBD patients. The diagnosis of IBS and IBD (including CD and UC) was based upon clinical assessment, questionnaire data, or specific symptom-based criteria, including the Manning and Rome criteria. Studies not reporting original data, manuscripts not published as full papers or those that did not use clinically validated methods to diagnose SIBO^17^ were excluded. Individuals in the control group included healthy asymptomatic controls as well as ‘patient controls’ including patients undergoing evaluation for unexplained ‘gastrointestinal syndromes’ (e.g., anemia, dyspepsia, pyrexia of unknown origin). PPI and antibiotic data were extracted from the selected studies. Eligibility criteria for study inclusion are provided in Table 1. Disagreements between reviewers were resolved by mutual consensus after reference to the original published paper.

**Table 1.** Eligibility criteria for the studies included in systematic review and meta-analysis

Eligibility criteria
Case-control or prevalence studies, published as full papers in peer reviewed journals.
Adults or children with a presumed diagnosis of Irritable bowel syndrome based on questionnaire, or meeting specific diagnostic criteria*. Adult patients with an established diagnosis of IBD (including CD and UC). Control group, referred to as ‘controls’ included ‘healthy asymptomatic controls’ as well as ‘patient controls’ including patients undergoing evaluation for unexplained gastrointestinal ‘syndromes’ (e.g., anemia, dyspepsia, pyrexia of unknown origin, diarrhea). Studies reporting on efficacy data after antibiotic treatment of methane positive SIBO in IBS and IBD patients was also included.
Clinically validated methods to diagnose SIBO**.
Participants not specially selected.
* Rome Criteria.^18–21^ ** Lactulose and/or Glucose breath test.

IBS: irritable bowel syndrome; IBD: inflammatory bowel disease; SIBO: small intestinal bacterial overgrowth; CD: Crohn’s disease; UC: ulcerative colitis.

### Data extraction and quality assessment

All data was extracted independently by two authors into a Microsoft Excel spreadsheet (2010 Professional edition; Microsoft Corp, Redmond, Washington, USA). During the data collection process, the following data was extracted from the studies; the author, the year of the study, country, source of controls, method of diagnosis of methane positive SIBO including test duration, quantity of substrate used and the cut off criteria for diagnosis of methane positive SIBO, gender, concurrent use of PPI and antibiotics, any significant co-morbidities including previous surgery for the patient and the control groups. In addition, for all patients with IBS and IBD, data regarding mode of diagnosis of IBS and IBD, sub-types, overlap with the other FGIDs, treatment of SIBO positive patients with antibiotics and objective and subjective response post treatment was recorded.

This systematic review and meta-analysis meets the preferred reporting items for systematic reviews and meta-analysis statement requirements (PRISMA).^22^ The quality of the included studies was assessed by using the Joanna Briggs Institute (JBI) critical appraisal tools for use in JBI systematic reviews for prevalence studies.^23^ The risk of bias was ranked as high when the study reached up to 49% of “yes” score, moderate when the study reached from 50 to 69% of “yes” score, and low when the study reached over 70% of “yes” score. In addition, the quality of the case-control included studies were assessed using the Newcastle-Ottawa scale (NOS) which judges the selection of the study groups, the comparability of the groups and the ascertainment of the exposure of interest, to assign a maximum score of 9 stars.^24^

### Data analysis

The initial step involved determining the number of cases with IBS and controls (using various diagnostic modalities) in the respective cohorts. The same was done in patients with IBD and controls. This was followed by calculating the pooled estimates of prevalence and odds ratios (OR) and 95% confidence intervals (CI) for the prevalence of methane positive SIBO in IBS and IBD patients with their respective controls. Subgroup analysis stratified by diagnostic modalities, IBS and IBD subtypes, quality of the studies as assessed utilizing the quality assessment tools NOS/JBI critical appraisal tool were conducted. We also summarized PPIs/antibiotics data that were reported in included case-control studies. Finally, we did sensitivity analysis including only high-quality studies (assessed utilizing the NOS/JBI critical appraisal tool), reporting the prevalence of methane positive SIBO in IBS and IBD patients with their respective controls.

Analyses for the association between methane positive SIBO and patients with IBS or IBD and descriptive analyses were carried out utilizing the Statistical Package for Social Sciences (SPSS Version 23, Armonk NY: IBM Corporation) and comprehensive Meta-analysis (CMS) Version 3.3.070. The major statistical method for this review would be pooled proportion meta-analysis (uses logit transformation of proportions) for prevalence and odds ratio for comparisons between IBS/IBD and controls. Pooled estimates of disease prevalence were calculated using a random effects model to appropriately account for variability in the summary estimate. Between study variation was evaluated using Cochrane’s test^25^ and was quantified through the I^2^ index in which values close to 100 indicate substantial variation between studies while values close to zero indicate minimal between-study variation. Standard approaches (Egger Test^26^ and inspection of Funnel Plots), were applied to identify potential publication biases. If one or more cells had a value of 0, then the CMS software automatically adds a fixed value of 0.5 to the respective cell for computation of log odds ratio and variance. Further, either Chi^2^ test *P* < .10 or I^2^ > 50% were taken as indications of substantial heterogeneity.

## Results

### Selection outcome

The initial search strategy identified 1,179 publications, but only 52 appeared to be relevant to the study question and were retrieved for further evaluation. Of these, 30 were excluded for various reasons, leaving 15 eligible IBS studies^12,27–40^ and 5 IBD studies^41–45^ and two studies^46,47^ reporting on prevalence rates of methane positive SIBO in both IBS and IBD patients (PRISMA flow diagram and Table S9). The characteristics of all the studies in the current meta-analysis including the methodology pertaining to diagnosis of SIBO and patient characteristics, are outlined in Table 2, Table 3 and Tables S1, S2, and S3. Seven of the 22 studies were conducted in USA,^27,28,33–35,39,40^ four each in India^12,29,36,44^and Italy,^32,37,41,42^ three in Korea,^31,43,48^ and one each in Spain,^38^ Brazil,^45^ Australia,^46^ and Israel.^47^ The studies from Israel and Australia looked at both IBS and IBD patients.

**Table 2.** Characteristics of studies showing mode of diagnosis and prevalence of methane positive SIBO in IBS patients

**No**	**Author**	**Study Year**	**Country**	**IBS, n**	**IBS Subtype** **IBS-D IBS-C IBS-M**			**Criteria for IBS diagnosis**	**Controls n**	**Type of control**	**Mode of diagnosis**	**Methane Positive SIBO in IBS, n (%)**	**Methane positive SIBO in IBS subtypes, n (%)** **IBS-D IBS-C IBS-M**			**Methane positive SIBO in controls, n (%)**
1	Shah A et al^46^	2020	Australia	62	18	8	38	Rome IV	63	*Healthy controls*	GBT	4 (25)	1 (6.3)	1 (6.3)	2 (12.5)	5 (31.3)
2	Ghoshal U et al^12^	2018	India	23	NA	23	-	Rome III	68	*Non-constipating IBS*	LBT	13 (56.5)	NA	13 (56.5)	-	25 (36.8)
3	Ghoshal U et al^29^	2016	India	25	13	12	NA	Rome III	NA	NA	LBT	8 (66.7)	3 (23.1)	8 (66.7)	NA	NA
4	Vega AB et al^38^	2015	Spain	48	NA	48	NA	Rome III	19	*Healthy controls*	LBT	29 (60.4)	NA	29 (60.4)	NA	10 (52.6)
5	Lee KN et al^48^	2013	Korea	68	35	23	10	Rome III	55	*Healthy controls*	LBT	10 (14.7)	21 (60)	14 (60.9)	7 (70)	5 (9.1)
6	Rana S et al^36^	2012	India	175	175	NA	NA	Rome II	150	*Healthy controls*	GBT	0	0	NA	NA	0
7	Park JS et al^31^	2010	Korea	76	45	12	19	NA	40	*Healthy controls*	LBT	25 (32.9)	14 (31.1)	4 (33.3)	7 (36.8)	13 (32.5)
8	Hwang L et al^39^	2010	USA	56	23	24	9	Rome I	NA	NA	LBT	28 (50)	6 (26.1)	22 (91.7)	-	NA
9	Parodi A et al^32^	2009	Italy	130	51	31	48	Rome III	70	*Healthy controls*	GBT	35 (26.9)	11 (21.6)	10 (32.3)	14 (29.2)	17 (24.3)
10	Scarpellini E et al^37^^	2009	Italy	43	15	12	16	Rome II	56	*Healthy controls*	LBT	4 (9.3)	-	-	-	0
11	Bratten J et al^27^	2008	USA	224	114	92	-	Rome II	40	*Healthy controls*	LBT	44 (19.6)	13 (11.4)	25 (27.2)	-	6 (15)
12	Majewski M et al^40^	2007	USA	204	149	30	25	Rome II	NA	NA	GBT	32 (15.7)	20 (13.4)	7 (23.3)	4 (16)	NA
13	Chatterjee S et al^28^	2007	USA	87	NA	NA	NA	Rome I	NA	NA	LBT	20 (23)	NA	NA	NA	NA
14	Pimentel M et al^33^	2006	USA	39	NA	39	NA	Rome I	NA	NA	LBT	12 (30.8)	NA	12 (30.8)	NA	NA
15	Pimentel M et al^34^	2003	USA	65	34	31	NA	Rome I	NA	NA	LBT	12 (24.0)	0	12 (38.7)	NA	NA
16	Pimentel M et al^35^	2003	USA	296	111	120	65	Rome I	NA	NA	LBT	50 (16.9)	6 (5.4)	30 (25)	NA	NA
17	Peled Y et al^47^	1987	Israel	32	6	16	-	Breath methane	152	*Healthy controls*	NA	11 (34.4)	1 (16.7)	5 (31.3)	-	76 (50)

IBS: Irritable bowel syndrome; SIBO: small intestinal bacterial overgrowth; LBT: lactulose breath test; GBT: glucose breath test; IBS-D: IBS-diarrhea; IBS-C: IBS-constipation; IBS-M: IBS-mixed; NA: not applicable, ^ Study included pediatric patients with a diagnosis of IBS, *Information not available.

**Table 3.** Characteristics of the IBD studies included in this systematic review and meta-analysis

Study No	Author	Study Year	Study Type	Country	IBD, n	UC, n	CD, n	Diagnostic criteria for IBD	Controls n	Type of control	Mode of diagnosis of SIBO	Methane positive SIBO in IBD, n (%)	Methane positive SIBO in UC, n (%)	Methane positive SIBO in CD, n (%)	Methane positive SIBO in Controls (%)
1	Shah A et al^46^	2020	Case control	Australia	81	48	33	Known IBD	63	Healthy controls	GBT	5 (31.3)	4 (25)	1 (6.3)	5 (31.3)
2	Ricci J et al^45^	2017	Case control	Brazil	92	NA	92	*IBD diagnosis	97	Nonspecific chronic GI symptoms	GBT	9 (9.8)	NA	9 (9.8)	8 (8.2)
3	Greco A et al^42^	2015	Prevalence	Italy	68	NA	68	Internationally accepted criteria	NA	NA	GBT	1 (1.5)	NA	1 (1.5)	NA
4	Lee JM et al^43^	2015	Case control	Korea	107	64	43	Known IBD	30	Healthy controls	GBT	3 (2.8)	-	-	0 (0)
5	Rana S et al^44^	2013	Case control	India	137	95	42	Confirmed by colonic biopsy	115	Healthy controls	GBT	4 (2.9)	-	-	28 (24.3)
6	Castiglione F et al^41^	2000	Case control	Italy	57	NA	57	Known IBD	40	Healthy controls	LBT	2 (3.5)	NA	2 (3.5)	0 (0)
7	Peled Y et al^47^	1987	Case control	Israel	84	51	33	*IBD diagnosis	152	Healthy controls	Breath methane was measured	18 (21.4)	16 (31.4)	2 (6.1)	76 (50)

IBD: inflammatory bowel disease; UC: ulcerative colitis; CD: Crohn’s disease; SIBO: small intestinal bacterial overgrowth; LBT: lactulose breath test; GBT: glucose breath test; GI: gastrointestinal; NA: not applicable; *IBD diagnosis: clinical, radiological, colonoscopy and histological diagnosis of IBD.

### IBS: Prevalence of methane positive SIBO

*All studies*: Overall, the 17 studies (10 case control studies^12,27,31,32,36–38,46–48^ and 7 prevalence studies^28,29,33–35,39,40^) assessing the prevalence of methane positive SIBO included 1,653 patients with IBS. The prevalence of methane positive SIBO in IBS patients was 25.0% (95% CI 18.8–32.4), Figure S1), with significantly high heterogeneity in the studies included in this analysis (I^2^=86.28, *p* = .0001). Visual inspection of the funnel plot revealed overall asymmetry, suggesting the potential for publication bias (Figure S2), consistent with the results of Egger’s test.

**Figure 1.**
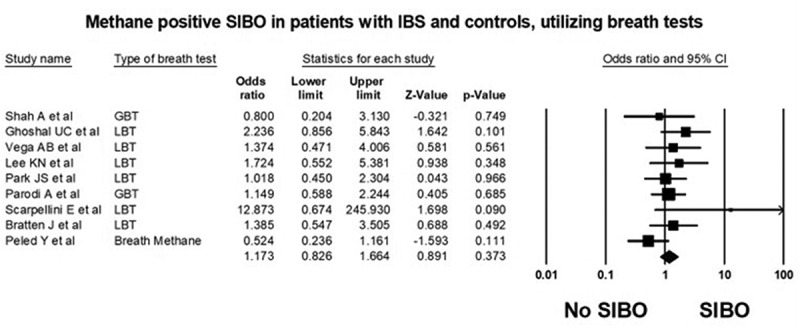
Forest plot of case-control studies showing methane positive SIBO in patients with IBS and controls, utilizing breath tests (OR = 1.2 (95% CI 0.8–1.7), *P* = .373), (I^2^=13.70, *P* = .320)

**Figure 2.**
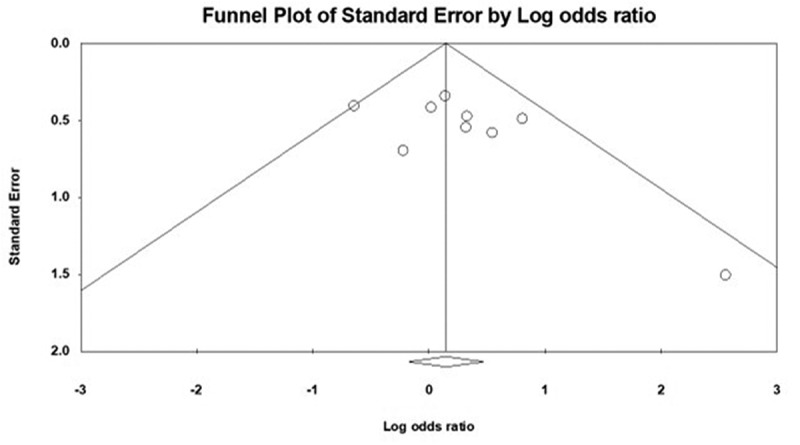
Funnel plot of methane positive SIBO in patients with IBS and controls

*Case control studies*: Ten^12,27,31,32,36–38,46–48^ of the 17 studies were case control studies and included 881 patients with IBS and 713 controls. The prevalence of methane positive SIBO in IBS patients (175/881, 19.95% [95% CI 17.3–22.7]) was not different from controls (157/713, 22.0% [95% CI 19.0–25.3]). Overall, the odds of methane positive SIBO was not statistically different in IBS patients as compared to controls (OR = 1.2 (95% CI 0.8–1.7), *P* = .373, Figure 1). There was no significant heterogeneity in the studies included in the analysis (I^2^=13.70, *P* = .320) and visual inspection of the funnel plot showed overall symmetry, suggesting minimal potential for publication bias, Figure 2.

**Influence of selection criteria for controls, and risk of bias on the prevalence of methane positive SIBO in patients with IBS and controls**

The quality of the included studies (all prevalence studies and the case group (only IBS patients) of the case control studies) as assessed by the JBI critical appraisal tool is shown in Table S6. Out of the ten case-control studies, eight studies^27,31,32,36–38,46,48^ presented a low risk of bias/high methodological quality, one^12^ (utilizing LBT for SIBO diagnosis) presented a moderate risk of bias/moderate methodological quality, and one^47^ (measuring breath methane) high risks of bias/low methodological quality. All seven prevalence studies^28,29,33–35,39,40^ presented a moderate risk of bias/moderate methodological quality. Furthermore, the majority (8/10, 80%) of the case-control studies were of high-quality, defined as a score of ≥6 using the Newcastle-Ottawa assessment scale (NOS), Table S5.

*High-quality studies with low risk of bias*: Including only studies with low risk of bias based upon the JBI critical appraisal tool and the NOS (supplementary material Tables S5 and S6), yielded a OR of 1.2 (95% CI 0.9–1.8, *P* = .246, Figure S3) for methane positive SIBO in IBS patients as compared to healthy asymptomatic controls. Furthermore, including only high-quality studies resulted in reducing heterogeneity to 0 (I^2^=0%, *P* = .744). Three^32,36,46^ out of the eight studies utilized GBT and the remaining five^27,31,37,38,48^ utilized LBT for diagnosis of methane positive SIBO in IBS patients and controls.

*Healthy controls*: If only studies^27,31,32,36–38,46–48^ where healthy asymptomatic controls were included, the OR for methane positive SIBO in IBS-patients was 1.1 (95% CI 0.8–1.5), *P* = .704, Figure S4). There was no significant heterogeneity (I^2^=3.303, *P* = .404) in the studies included in this analysis. This analysis excluded only one study,^12^ with non-constipating IBS as controls.

**Comparison of methane positive SIBO in IBS according to the type of breath test utilized for SIBO diagnosis.**

Twelve studies^12,27–29,31,33–35,37–39,48^ utilized LBT and four studies^32,36,40,46^ utilized GBT for SIBO diagnosis. One study^47^ measured breath methane and considered a subject methane producer if the breath methane concentration was at least 1 parts per million (ppm)above ambient air. The overall prevalence of methane positive SIBO in IBS was almost 3-fold higher for studies utilizing LBT (29.0% (95% CI 20.9–38.6), Figure S5), as compared to studies utilizing GBT (11.5% (95% CI 5.0–24.3), fFigure S7). There was significantly high heterogeneity in both these analysis (I^2^=87.10, *P* = .0001 for LBT and 86.32, *P* = .0001 for GBT). Visual inspection of the funnel plot revealed asymmetry, suggesting publication bias, Figure S6.

The OR for methane positive SIBO in IBS of 1.5 (95% CI 1.0–2.3), *P* = .06, Figure S8) for studies using LBT was higher as compared to 1.1 (95% CI 0.6–2.0), *P* = .824, Figure S9) for studies utilizing GBT. Moreover, there was no significant heterogeneity in the studies utilizing LBT (I^2^=0, *P* = .590) or GBT for diagnosing methane positive SIBO in IBS (I^2^=0, *P* = .641).

### IBS subtypes and prevalence of methane positive SIBO

Eleven^27,31,32,34,35,39,40,46–49^ out of 17 studies reported on methane positive SIBO in IBS-subtypes (Table 2). Of these, only five studies^31,32,40,46,48^ reported prevalence rates of methane positive SIBO in all distinct IBS-subtypes. The prevalence of SIBO was higher in patients with IBS-C at 37.7% (95% CI 33.5–42.1) as compared to 24.3% (95% CI 17.4–32.3) in IBS-M and almost 3-fold higher as compared to patients with IBS-D 12.4% (95% CI 10.2–14.9). The odds of methane positive SIBO in IBS-C was significantly higher as compared to IBS-D, (OR = 3.1 (95%CI 1.7–5.6), *P* = .0001, Figure S10) with substantial heterogeneity in the included studies (I^2^=52.23, *p* = .02). However, including only high-quality studies the prevalence of methane positivity on breath test remained significantly higher in IBS-C as compared to IBS-D (OR = 2.0, (95% CI 1.3–3.2),*P* = .002), and there was no significant heterogeneity in this analysis (I^2^=0, *P* = .540). There was no significant difference in the odds of methane positive SIBO in IBS-C as compared to IBS-M or IBS-D as compared to IBS-M (data not shown).

### Association between methane positive SIBO in patients with IBS and oro-cecal transit time

Only two^12,38^ out of 17 studies included in this systematic review and meta-analysis reported on the intestinal transit time in methane producers and compared it to that in non-methane producers. Vega et al,^38^ reported a significantly longer oro-cecal transit time (OCTT) in both healthy controls and constipated, methane-producing subjects as compared to non-methane producers. Similarly, Ghoshal et al,^12^ reported longer OCTT in methane producers as compared to non-methane producers, however it was not significant (*P* = .06).

### Effect of proton pump inhibitors on the prevalence of methane positive SIBO in IBS

Overall, only one^29^ out of 17 studies included IBS patients who were not on PPI therapy. 15 studies^12,27,28,31–40,47,48^ were excluded because data about effect of PPI on methane positivity on breath test in IBS patients could not be extracted, Table S1. Only one case control study^46^ assessed the effect of PPI therapy on methane positivity in IBS patients. The prevalence of methane positive SIBO was higher in 3/26 (11.5%, 95% CI 2.4–30.1) IBS patients on PPI as compared to those 2/36 (5.5%, 95% CI 0.6–18.6) not on a PPI, a non-significant difference.

### Effect of antibiotic therapy on the prevalence of methane positive SIBO in IBS

Only three^12,33,38^ out of 17 studies included in this systematic review and meta-analysis, assessed the effect of treatment (one study of rifaximin^12^ and one study of neomycin,^33^ and one study with fiber^38^) on symptom improvement in methane positive IBS-C patients, Table S4. While studies could not be combined for meta-analysis due to markedly different treatment modalities (type of antibiotic, fiber), all three studies demonstrated that the treatment of methane positive IBS-C patients with antibiotics/fiber (as compared to placebo) resulted in symptom improvement and improvement or normalization of follow-up breath tests in a large proportion of treated patients.

### IBD: Prevalence of methane positive SIBO

*All studies*: In total, seven studies assessed the prevalence of methane positive SIBO in 626 adult patients with IBD. The prevalence of methane positive SIBO in patients in IBD patients was 5.6% (95% CI 2.6–11.8, Figure S11). Substantial heterogeneity was noted in the overall analysis, (I^2^=80.4, *p* = .0001). Five studies utilized GBT^42–46^ and only one study utilized LBT.^41^ One study^47^ measured breath methane and considered a subject a methane producer if the breath methane concentration was at least 1 ppm above ambient air. Hence, we did not do a subgroup analysis according to the type of breath test utilized to diagnose SIBO in patients with IBD.

**Figure 3.**
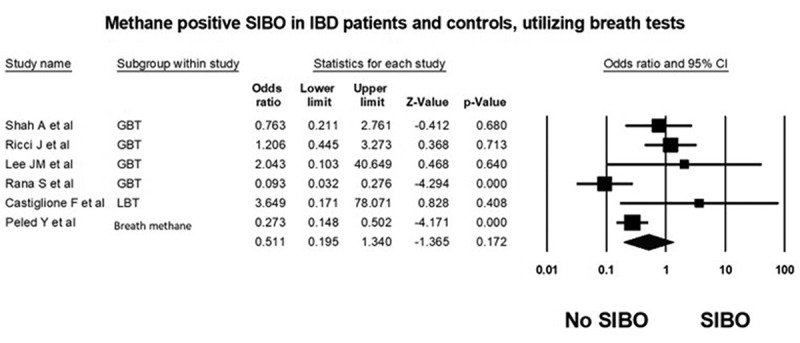
Forest plot of case control studies showing prevalence of methane positive SIBO in patients with IBD and controls, utilizing breath tests (OR = 0.5 [95% CI 0.2–1.3], *P* = .17), (I^2^=70.8, *P* = .004)

*Case control studies*: Six^41,43–47^ of the of the seven studies were case control studies and included 558 adult patients with IBD and 497 controls. The prevalence of methane positive SIBO in patients with IBD was approximately 3-fold lower at 7.4% (95% CI 5.4–9.8) compared to 23.5% (95% CI 19.8–27.5) in controls. Overall analysis comprising all patients with IBD (including patients with CD and UC) found no difference in the odds of methane positive SIBO in IBD (OR = 0.5 (95% CI 0.2–1.3, *P* = .172 Figure 3), with substantial heterogeneity detected between the studies included in this analysis (I^2^=70.8, *P* = .004).

**Influence of selection criteria for controls, and risk of bias, type of breath test on the prevalence of methane positive SIBO in patients with IBD and controls**

The quality of the included studies based on the JBI critical appraisal tool and the NOS is shown in Tables S7 and S8. Out of the seven studies, five studies presented a low risk of bias/high methodological quality and two studies (one utilizing GBT^45^ and one measured breath methane^47^) presented high risk of bias/low methodological quality.

*High-quality studies with low risk of bias*: Including only five studies^41–44,46^ with low risk of bias based upon the quality assessment tools (supplementary material Tables S7 and S8), 5 high-quality studies, yielded a significantly low prevalence for methane positive SIBO at 3.6% (95% CI 2.2–6.0, Figure S12) in patients with IBD. There was no heterogeneity in this subgroup analysis (I^2^=0, *P* = .595). Conducting a sensitivity analysis with studies utilizing GBT, the results remained unchanged (data not shown).

### Methane positive SIBO in IBD subtypes

In regard to IBD subtypes, three^41,42,45^ out of seven studies included patients with CD, while four included both UC and CD patients^43,44,46,47^ (all case control studies). Data from two out the four studies^43,44^ could not be extracted due to insufficient information on methane positive SIBO in IBD subtypes. Methane positive SIBO was approximately 4-fold higher in UC patients at 20.2% (95% CI 12.8–29.4) as compared to 5.3% (95% CI 3.0–8.5) in CD patients, Table 3.

### Assessment of risk factors for methane positive SIBO in IBD

None of the studies reported on the influence of risk factors such as disease duration, prior surgery, fibro-stenosing disease, location of IBD, and the influence of immunotherapy on methane positive SIBO in IBD patients, hence we were unable to conduct a subgroup analyses to assess the impact of risk factors of methane positivity on breath test in IBD patients. With regards to transit time, Rana et al,^50^ did not find any significant difference in the oro-cecal transit time measured utilizing LBT between methane positive IBD patients (145.2 ± 28.7) as compared to methane negative IBD patients (117.7 ± 33.45). This association between transit time and methane was not reported in the remaining studies included in this analysis.

**Effect of antibiotic therapy and proton pump inhibitors on the prevalence of methane positive SIBO in IBD**

We found no studies that directly evaluated the effect antibiotic treatment has on the prevalence of methane positive SIBO in IBD. Four^41,43–45^ out of seven studies, included IBD patients that were not on a PPI and in two^42,47^ out of seven studies information about PPI use was not available, (Table S2). Only one case control study^46^ assessed the effect of PPI therapy on methane positive SIBO in IBD patients. The prevalence of methane positive SIBO was higher in IBD patients on PPI (15.4%, 95% CI 1.9–45.4) as compared to patients not on PPI (4.4%, 95% CI 9.2–12.3).

## Discussion

This is the first systematic review and meta-analysis of methane positive SIBO in patients with IBS and IBD and is based on 22 peer-reviewed case control and prevalence studies from eight different countries that included 1,653 patients with IBS, 626 patients with IBD, and 1,210 controls. We found patients with IBS-C have a significantly higher prevalence of methane positivity on breath tests as compared to those with IBS-D (OR = 3.1, 95% CI 1.7–5.6). In contrast, if IBS patients are not differentiated according to their subtype, the prevalence of methane positive SIBO is not increased in IBS as compared to controls (OR = 1.2, 95% CI 0.8–1.7). Methane positive SIBO was not associated with IBD (OR 0.5, 95% CI 0.2–1.3), although patients with UC have a significantly greater prevalence of breath methane positivity as compared to those with CD. The primary analysis (including both prevalence and case-control studies) reporting methane positive SIBO in IBS and IBD patients revealed substantial heterogeneity. This could at least partially be explained by the inherent limitations of the studies and the limitations of the available tests for SIBO diagnosis. Hence, we conducted subgroup analysis, according to the study type, type of controls, the quality of the studies as assessed utilizing the quality assessment tools (NOS and JBI appraisal tool), and the type of breath test used for SIBO diagnosis.

In these analyses we found very little or even no heterogeneity and the prevalence rates for methane positive SIBO was not higher in IBS patients as compared to controls. Importantly, the majority (8/10) of the case control studies scored high on the quality assessment tools, while all prevalence studies had a moderate risk of bias. Finally, conducting subgroup analysis according to the type of breath test, we found a 3-fold (significantly) increased prevalence of methane positive SIBO in IBS patients in studies utilizing LBT as compared to those utilizing GBT, with significant heterogeneity in both analyses. On the other hand, although the OR for methane positive SIBO in IBS patients was significantly higher in case-control studies utilizing LBT as compared to those utilizing GBT, there was no heterogeneity in these analyses. This suggests that the type of study (prevalence vs. case-control) rather than the type of breath test used for SIBO diagnosis, contributed to the high heterogeneity in the primary analyses.

Next, we examined studies reporting methane positive SIBO in IBD patients. Due to the limited number of studies, we were unable to make comparisons on methane prevalence rates in IBD patients according to the type of study or type of breath test. However, including only high-quality studies (5/7), we found significantly lower prevalence rates for methane positive SIBO in IBD patients and there was no heterogeneity in studies included in this analysis.

One of the important findings of this systematic review and meta-analysis is the significant association between methane on breath tests and IBS- and IBD subtypes. We found a 3-fold higher prevalence of methane positive SIBO in patients with IBS-C as compared to those with IBS-D, but there was substantial heterogeneity in the analysis. However, conducting subgroup analysis, including only high-quality studies, the odds of methane positive SIBO were still significantly higher in IBS-C as compared to IBS-D (OR 2.0, 95% CI 1.3–3.2) and importantly the heterogeneity in this analysis was reduced to zero. Our findings were in keeping with those by Kunkel et al,^11^ who reported a significantly increased prevalence of methane positive SIBO in patients with IBS-C and functional constipation, but also noted very high heterogeneity in the studies included in the analysis. Only two studies included in this systematic review and meta-analysis reported on oro-cecal transit time and methane. Both found longer oro-cecal transit time in methane producers as compared to non-methane producers. There is experimental^51^ and clinical evidence that methane is likely capable of slowing intestinal transit,^11,52^ implicating either a direct or indirect action of methane inducing constipation. Analyzing stool microbiome utilzing qPCR, Kim et al^53^ and more recently Ghoshal et al,^29^ demonstrated that *Methanobrevibacter smithii* is more abundant in methane producing IBS-C subjects as compared to non-methane producing IBS-C patients. Interestingly, they also demonstrated a correlation between the concentration of *M. smithii* and the functional consequence of their metabolic activity, i.e., quantity of methane in exhaled air. However, other human studies have shown that slow intestinal trasit may facilitate the growth of methanogenic bacteria.^47,54^ Thus, the question does methane slow the transit time or does a delayed transit time promotes the growth of methanogens (hence the production of methane) remains to be elucidated.

Although only limited data are available, we found a significantly (almost 4-fold) lower prevalence of methane positive SIBO in patients with CD (5.3%) as compared to those with UC (20.2%). More importantly, only one^50^ out the seven studies included in the meta-analysis assessed the transit time in IBD patients. This study did not find any significant association between oro-cecal transit time and methane status in IBD patients. These findings are intriguing, as in our recent systematic review and meta-analysis,^16^ we found a significantly a higher prevalence of non-methanogenic SIBO utilizing breath testing in IBD patients (and higher in patients with CD as compared to UC) versus non-IBD controls (OR 9.5, 95% CI 3.39–26.68). Moreover, the oro-cecal transit time was prolonged in IBD patients compared to healthy controls, and the oro-cecal transit time was significantly increased in SIBO positive compared to SIBO negative IBD patients. Collectively, these findings suggest that breath methane, methanogen positivity as measured by PCR/culture and OCTT are not inextricably linked, and that methanogen persistence is differentially affected by other alterations to the nutritional and/or environmental landscape of the gut milieu in CD and UC.

One of the key limitations of this systematic review is the small number of studies measuring both, methane and hydrogen measurements during breath tests. We did not include conference abstracts, since the brevity of abstracts frequently fails to provide adequate information to appropriately appraise the design, methods, risk of bias, and outcomes of the studies included in the abstracts. Balancing the risk of publication bias against potentially misleading data, we opted to include only fully peer-reviewed published manuscripts. Indeed, a recent study comparing abstracts with full-length journal articles concluded that the information presented in abstracts was not dependable^55^ Attempts were made to contact authors of the studies included in this systematic review and meta-analysis to get additional information and/or clarification whenever this deemed necessary. Two research groups responded, and the additional information was incorporated in the analyses. Even though this systematic review and meta-analysis has not been prospectively registered, we acknowledge that prospective registration of systematic reviews is now recommended since it promotes transparency, reduces potential for bias and avoids duplication of reviews.^56^

Although there more than 60 peer-reviewed published studies (both case-control and prevalence studies) assessing the link between SIBO and IBS and IBD, less than half have measured both, methane and hydrogen on breath testing. It is now well acknowledged that lack of measurement of methane on routine breath testing could underestimate SIBO prevalence in various gastrointestinal conditions.^14^ Another important limitation is that all studies included in this systematic review and meta-analysis have only utilized breath tests (indirect testing), which are surrogate markers for diagnosing bacterial overgrowth. Methanogens are strict anaerobes^57^ and are exceedingly difficult to culture, requiring very specific conditions and culture media.^58^ Moreover, currently there is no consensus regarding the gold standard for the diagnosis of SIBO. While direct (small bowel aspirate and culture) tests are invasive, time consuming and require an endoscopy, they have been largely replaced in clinical practice by (indirect) breath tests. However, compared with culture-based methods, the GBT has a sensitivity of 62.5% and a specificity of 81.7% and the LBT has a sensitivity of 52.4% and a specificity of 85.7%.^59^Molecular techniques (PCR-based tests), utilizing specific primers to quantify small intestinal bacterial colonization are emerging as alternative diagnostic approaches.^46^

The assessment of the link between methanogens and IBS or IBD is further limited by the paucity of studies assessing the effect of antibiotic and PPI use specifically on methane positive SIBO in these patients. It is now well established that PPI is a risk factor for SIBO^60^ in various gastrointestinal disorders. Proton pump inhibitors cause chronic acid suppression and the resultant hypochlorhydria alters the intraluminal environment to promote growth of the microbes in the small intestine. Only one study^46^ specifically assessed the effect of PPI on methane positive SIBO in IBS and IBD and found a higher prevalence rate of methane positive SIBO in both IBS and IBD patients on a PPI, as compared to those not on a PPI.

Due to the limited number of studies, it was not possible to conduct subgroup analysis to assess the effect of antibiotic therapy on symptom improvement, effect on transit time and normalization of follow-up breath tests in patients with IBS and IBD. Ghoshal et al,^12^ in their recent randomized controlled trial showed in thirteen methane producers, treatment with rifaximin as compared to placebo resulted in significant improvement in weekly stool frequency (*P* = .05), stool form (*P* = .08) and area under the curve (AUC) for methane on post treatment breath test (*P* = .005). Similarly, Pimentel et al^33^ showed that in twelve methane positive IBS-C patients, treatment with neomycin as compared to placebo was associated with significant improvement in constipation and normalization of methane on follow-up breath test in those treated with neomycin. In an elegant study by Vega et al,^38^ treatment of constipated patients (IBS-C and functional constipation) with fiber was associated with a significant improvement in the colonic transit time, symptoms and decreased methane production. This may support the concept that slow transit causes methane positivity but is not definitive.

In summary, this is the first systematic review and meta-analysis specifically examining the prevalence of methane positive “SIBO” or “IMO” in patients with IBS and IBD (Table 4). Based on the available data the prevalence of methane positive SIBO is increased in patients with IBS-C and decreased in patients with IBD, importantly in those with CD. Although PPI appeared to be a risk factor for methane positive SIBO in IBS and IBD patients, and antibiotic therapy was associated with improvement in symptoms and transit time, these data is extremely limited and need validation in larger studies. All this emphasizes the importance of measuring methane in addition to hydrogen on routine breath tests and the need for more robust tests (including isolation and culturing the fastidious methanogens) or utilizing molecular techniques (PCR-based techniques) to diagnose small intestinal methanogen overgrowth. While this systematic review and meta-analysis suggests a link between methane positive SIBO and gastrointestinal disorders, the quality of evidence is low, and this can be attributed mainly to substantial clinical heterogeneity seen in the prevalence studies. Thus, more appropriately designed studies are required that not only assess the prevalence of SIBO but also characterize intestinal dysbiosis in various gastrointestinal disorders.

**Table 4.** Summary of findings of the outcomes reported in this systematic review and meta-analysis

Mode of diagnosis of SIBO in IBS/IBD	No of studies	IBS/IBD n	Controls n	SIBO in IBS /IBD patients n	SIBO in Controls n	Prevalence rates of SIBO in IBS/IBD patients, % (95% CI)	Prevalence rates of SIBO in controls, % (95% CI)	Prevalence of SIBO, OR (95% CI)	Assessment of heterogeneity between studies
**Irritable Bowel Syndrome**									
All studies	17	1653	-	340	-	25.0 (18.8–32.4)*	-	***P* = .825**	I^2^=86.28, *p* = .0001
Only high-quality studies (All were case control studies)	8	826	493	151	56	18.3 (15.7–21.1)	11.4 (8.7–14.5)	1.2 (0.9–1.8), *p* = .246	I^2^=0, *p* = .744
Case control studies ONLY	10	881	713	175	157	19.9 (17.3–22.7)	22.0 (19.0–25.3)	1.2 (0.8–1.7), *p* = .373 ***P* = .124**	I^2^=13.7, *p* = .320
Case control studies including only healthy controls	9	858	645	162	132	18.9 (16.2–21.7)	20.5 (17.4–23.8)	1.1 (0.8–1.5), *p* = .704	I^2^=3.30, *p* = .404
Case control studies utilizing LBT	6	482	278	125	59	25.9 (22.7–30.1)	21.2 (16.6–26.5)	1.5 (1.0–2.3), *p* = .06	I^2^=0, *p* = .59
Case control studies utilizing GBT	3	367	283	39	22	10.6 (7.7–14.2)	7.8 (4.9–11.5)	1.1 (0.6–2.0), *p* = .824	I^2^=0, *p* = .641
IBS subtypes (IBS-C versus IBS-D)	11	509 (IBS-C)	774 (IBS-D)	192 (IBS-C)	96 (IBS-D)	37.7 (33.5–42.1)	12.4 (10.2–14.9)	3.1 (1.7–5.6), *p* = .0001 ***P* = .314**	I^2^=52.23, *p* = .02
**Inflammatory Bowel Disease**									
All studies	7	626	-	42	-	5.6 (2.6–11.8)*	-	-	I^2^=80.4, *p* = .0001
Case control studies ONLY	6	558	497	41	117	7.4 (5.4–9.8)	23.5 (19.8–27.5)	0.5 (0.2–1.3), *p* = .172-	I^2^=70.8, *p* = .004
CD	2	283	-	15	-	5.3 (3.0–8.5)	-		-
UC	2	99	-	20	-	20.2 (12.8–29.4)	-	-	-
Only high-quality studies (4 case control & 1 cohort study)	5	450	-	15	-	3.6 (2.2–6.0)*	-	-	I^2^=0, *p* = .595

IBS: Irritable bowel syndrome; IBS-D: IBS-diarrhea; IBS-C: IBS-constipation; IBD: inflammatory bowel disease; UC: ulcerative colitis; CD: Crohn’s disease; SIBO: small intestinal bacterial overgrowth; LBT: lactulose breath test; GBT: glucose breath test; OR: odds ratio; *pooled prevalence rate of SIBO in respective GI conditions calculated utilizing the CMA software, **P** indicates values from Eggers’s test.

## Supplementary Material

Supplemental Material
