# Supplementary material for: Methane positive small intestinal bacterial overgrowth in inflammatory bowel disease and irritable bowel syndrome: A systematic review and meta-analysis
Source: Gut Microbes. 2021 Jun 30;13(1):1933313. doi: 10.1080/19490976.2021.1933313 (PMC8253120; doi:10.1080/19490976.2021.1933313)
Supplement: Supplemental Material [file KGMI_A_1933313_SM7036.docx]

**Supplementary Material for manuscript:** Methane positive small intestinal bacterial overgrowth in inflammatory bowel disease and irritable bowel syndrome: A systematic review and meta-analysis

**Legend of Figures:**

**Figure S1:** Forest plot of studies showing methane positive SIBO in patients with IBS, utilizing breath tests (25.0% (95%CI 18.8-32.4)), (I^2^=86.28, p=0.0001).

**Figure S2:** Funnel plot of methane positive SIBO in patients with IBS, utilizing breath test.

**Figure S3:** Forest plot of case control studies showing prevalence of methane SIBO in patients with IBS, when only high-quality studies are included (OR=1.2 (95%CI 0.9-1.8), p=0.246), (I^2^=0, p=0.744).

**Figure S4:** Forest plot of case control studies showing prevalence of methane SIBO in patients with IBS, and healthy controls (OR=1.1 (95%CI 0.8-1.5), p=0.704), (I^2^=3.30, p=0.404).

**Figure S5:** Forest plot of studies showing methane positive SIBO in patients with IBS, utilizing lactulose breath test (29.0% (95%CI 20.9-38.6)), (I^2^=87.10, p=0.0001).

**Figure S6:** Funnel plot of methane positive SIBO in patients with IBS, utilizing lactulose breath test.

**Figure S7:** Forest plot of studies showing methane positive SIBO in patients with IBS, utilizing glucose breath tests (11.5% (95%CI 5.0-24.3)), (I^2^=86.32, p=0.0001).

**Figure S8:** Forest plot of case control studies showing prevalence of methane positive SIBO in patients with IBS and controls, utilizing lactulose breath test (OR=1.5 (95%CI 1.0-2.3), p=0.06), (I^2^=0, p=0.59).

**Figure S9:** Forest plot of case control studies showing prevalence of methane positive SIBO in patients with IBS and controls, utilizing glucose breath test (OR=1.1 (95%CI 0.6-2.0), p=0.824), (I^2^=0, p=0.641).

**Figure S10:** Forest plot of case control studies of methane positive SIBO in IBS subtypes (IBS D versus IBS C), (OR=3.1(95%CI 1.7-5.6), p=0.0001), (I^2^=52.23 p=0.02).

**Figure S11:** Forest plot of studies showing methane positive SIBO in patients with IBD, utilizing breath tests (5.6% (95%CI 2.6-11.8)), (I^2^=80.4, p=0.0001).

**Figure S12:** Forest plot of studies showing methane positive SIBO in patients with IBD, in high quality studies utilizing breath tests (3.6% (95%CI 2.2-6.0)), (I^2^=0, p=0.595).

**Figure S13:** Search strategy for MEDLINE**.**

**Legend of Tables:**

**Table S1:** Assessment of risk factors for methane positive SIBO in IBS patients included in this systematic review and meta-analysis.

**Table S2:** Assessment of risk factors and cut off criteria for methane positive SIBO in IBD patients included in the systematic review and meta-analysis.

**Table S3:** Assessment of cut off criteria for diagnosing methane positive SIBO in IBS patients.

**Table S4:** Studies evaluating the effect of antibiotic treatment in IBS patients with methane positive SIBO.

**Table S5:** Newcastle-Ottawa scale for assessment of quality of case control studies assessing the prevalence of methane positive SIBO in patients with IBS included in the systematic review and meta-analysis.

**Table S6:** Joanna Briggs Institute (JBI) Critical Appraisal Tools for quality assessment of prevalence studies and the case group of the case-control studies assessing methane positive SIBO in IBS included in the systematic review and meta-analysis.

**Table S7:** Newcastle-Ottawa scale for assessment of quality of case control studies assessing the prevalence of methane positive SIBO in patients with IBD included in the systematic review and meta-analysis.

**Table S8:** Joanna Briggs Institute (JBI) Critical Appraisal Tools for quality assessment of prevalence studies and the case group of the case-control studies assessing methane positive SIBO in IBD included in the systematic review and meta-analysis.

**Table S9:** Exclusion criteria for studies excluded from the systematic review and meta-analysis.

**Figure S1:** Forest plot of studies showing methane positive SIBO in patients with IBS, utilizing breath tests (25.0% (95%CI 18.8-32.4)), (I^2^=86.28, p=0.0001).

**Figure S2:** Funnel plot of methane positive SIBO in patients with IBS, utilizing breath test.


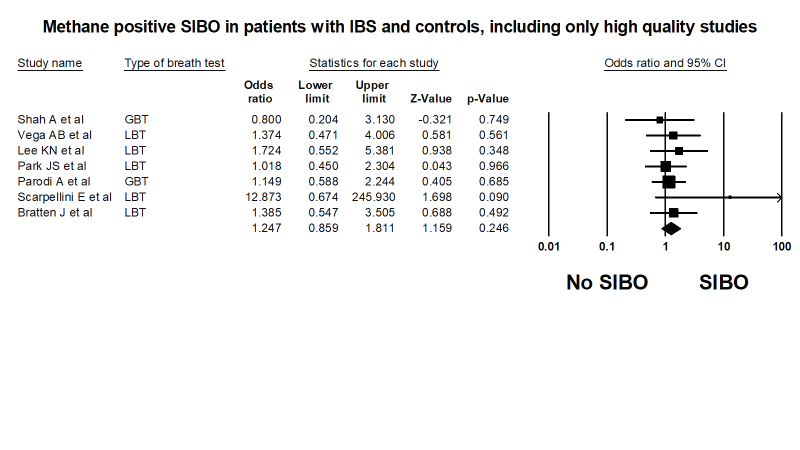


**Figure S3:** Forest plot of case control studies showing prevalence of SIBO in patients with IBS, when only high-quality studies are included (OR=1.2 (95%CI 0.9-1.8), p=0.246), (I^2^=0, p=0.744).


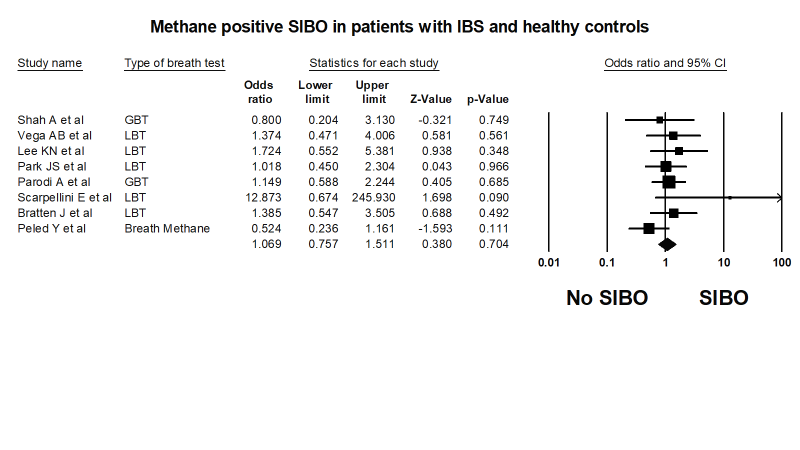


**Figure S4:** Forest plot of case control studies showing prevalence of SIBO in patients with IBS, and healthy controls (OR=1.1 (95%CI 0.8-1.5), p=0.704), (I^2^=3.30, p=0.404).


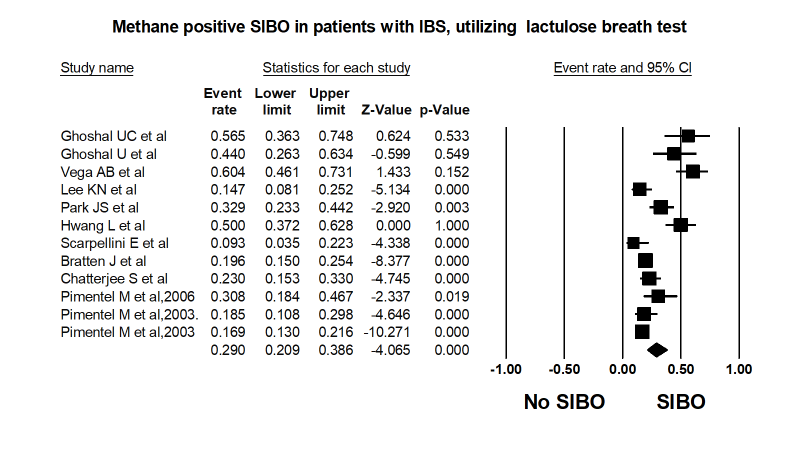


**Figure S5:** Forest plot of studies showing methane positive SIBO in patients with IBS, utilizing lactulose breath test (29.0% (95%CI 20.9-38.6)), (I^2^=87.10, p=0.0001).

**Figure S6:** Funnel plot of methane positive SIBO in patients with IBS, utilizing lactulose breath test.


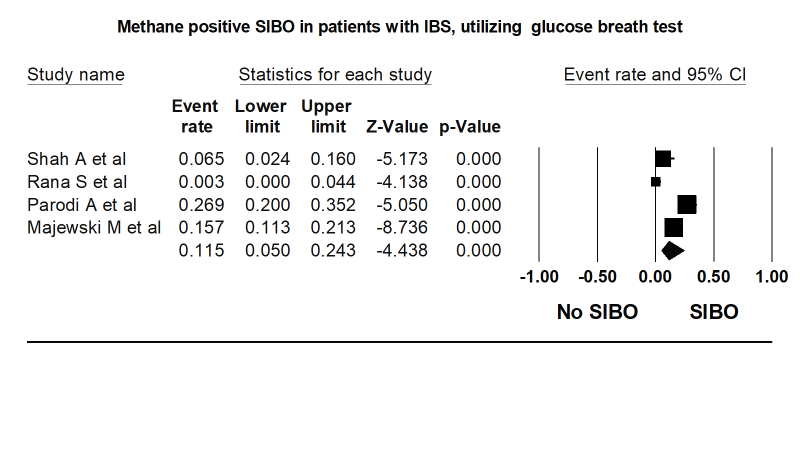


**Figure S7:** Forest plot of studies showing methane positive SIBO in patients with IBS, utilizing glucose breath tests (11.5% (95%CI 5.0-24.3)), (I^2^=86.32, p=0.0001).

**
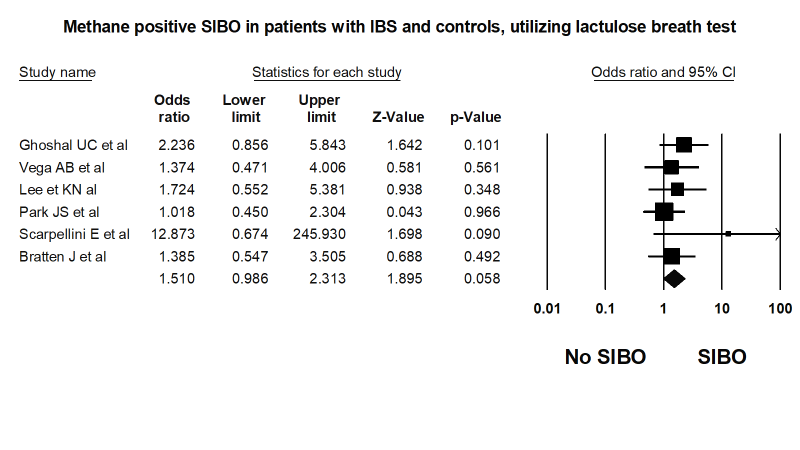
**

**Figure S8:** Forest plot of case control studies showing prevalence of methane positive SIBO in patients with IBS and controls, utilizing lactulose breath test (OR=1.5 (95%CI 1.0-2.3), p=0.06), (I^2^=0, p=0.59).


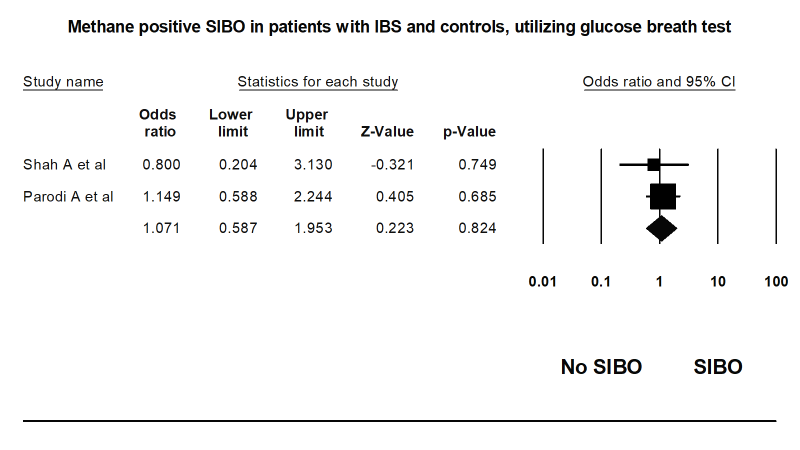


**Figure S9:** Forest plot of case control studies showing prevalence of methane positive SIBO in patients with IBS and controls, utilizing glucose breath test (OR=1.1 (95%CI 0.6-2.0), p=0.824), (I^2^=0, p=0.641).


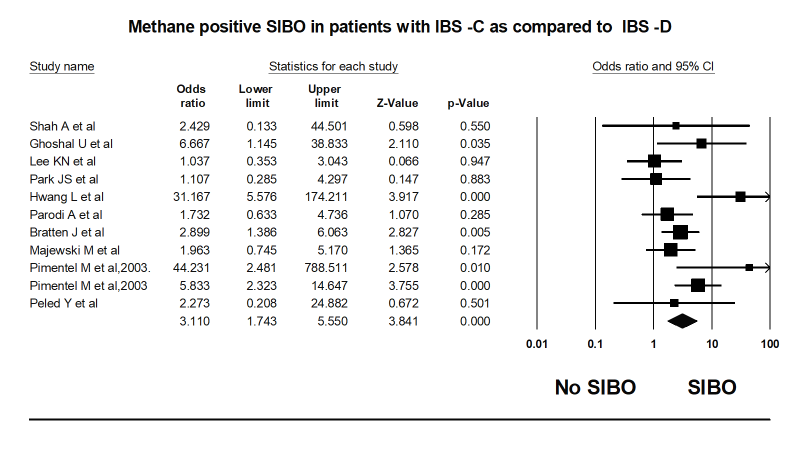


**Figure S10:** Forest plot of case control studies of methane positive SIBO in IBS subtypes (IBS D versus IBS C), (OR=3.1(95%CI 1.7-5.6), p=0.0001), (I^2^=52.23 p=0.02).


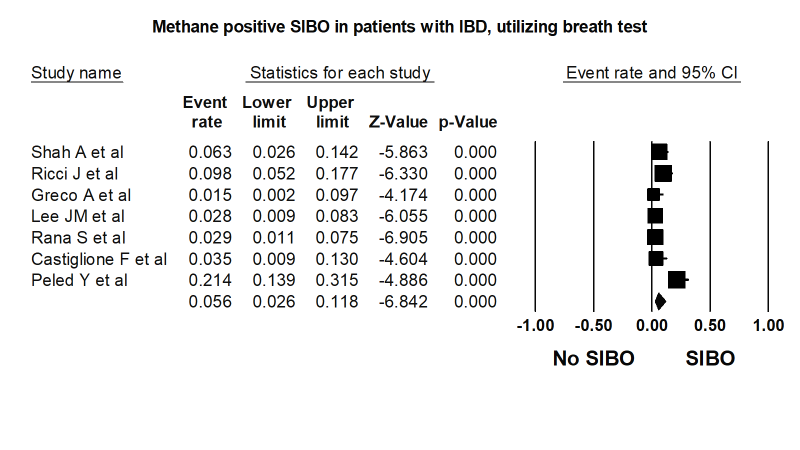


**Figure S11:** Forest plot of studies showing methane positive SIBO in patients with IBD, utilizing breath tests (5.6% (95%CI 2.6-11.8)), (I^2^=80.4, p=0.0001).


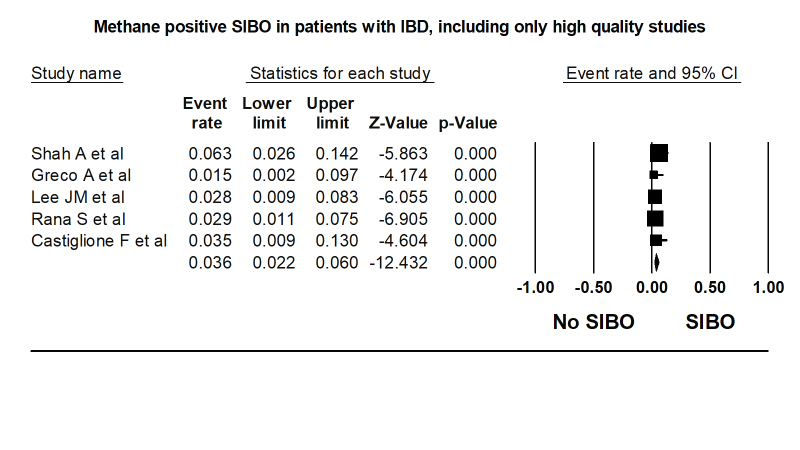


**Figure S12:** Forest plot of studies showing methane positive SIBO in patients with IBD, in high quality studies utilizing breath tests (3.6% (95%CI 2.2-6.0)), (I^2^=0, p=0.595).

| **Database search strategy MEDLINE(PubMed)** |
| --- |
| 1. methane[tw] OR CH4[tw]  2. “breath test”[tw] OR “breath tests”[tw] OR “breath testing”[tw] OR “breathalyzer tests”[tw] OR “breathalyzer test”[tw] OR “breathalyzer testing”[tw] OR “breath analysis” [tw]  3. constipation[tw] OR “colonic inertia” [tw] OR transit[tw] OR motility[tw] OR “irritable bowel syndrome” [tw] OR “irritable bowel syndromes” [tw] OR IBS [tw]  4. ‘’inflammatory bowel disease’’[tw] OR IBD [tw] OR “ulcerative colitis” [tw] OR “crohn’s disease” [tw]  5. #1 AND #2 AND #3 AND #4  *Text Word [tw] = search in following fields, title, abstract, MeSH headings and subheadings, author supplied keywords, substances.* |

**Figure S13:** Search strategy for MEDLINE**.**

**Table S1:** Assessment of risk factors for methane positive SIBO in IBS patients included in this systematic review and meta-analysis.

| **No** | **Author** | **Prior antibiotic use** | **Concurrent PPI** | **Treatment with antibiotic** | **Duration of treatment** | **Treatment efficacy** | **Prior Surgery** |
| --- | --- | --- | --- | --- | --- | --- | --- |
| 1 | Shah A et al^1^ | No | 26/62 were on PPI | No | NA | NA | NA |
| 2 | Ghoshal UC et al^2^ | None for the past 3 months | NA | Rifaximin | 1 month | methane was lower in patients on rifaximin vs placebo | NA |
| 3 | Ghoshal U et al^3^ | None for the past 1 month | No | No | NA | NA | No |
| 4 | Vega A et al^4^ | None for the past 3 months | NA | No | NA | NA | No |
| 5 | Lee KN et al^5^ | None for the past 3 months | NA | No | NA | NA | No |
| 6 | Rana S et al^6^ | None for the past 1 month | NA | No | NA | NA | No |
| 7 | Park JS et al^7^ | NA | NA | NA | NA | NA | NA |
| 8 | Hwang L et al ^8^ | NA | NA | NA | NA | NA | No |
| 9 | Parodi A et al^9^ | None for the past 2 weeks | NA | NA | NA | NA | NA |
| 10 | Scarpellini E et al^10^ | None for the past 2 months | NA | No | NA | NA | No |
| 11 | Bratten JR et al^11^ | None for the past 1 month | NA | No | NA | NA | No |
| 12 | Majewski M et al^12^ | None for the past 1 month | Yes, 45/93 (45.6%) GBT positive IBS patients were on PPI | Rifaximin | 1 month | Not specific for methane | NA |
| 13 | Chatterjee S et al^13^ | None for the past 3 months | NA | No | NA | NA | No |
| 14 | Pimentel M et al^14^ | None for the past 3 months | NA | Neomycin | 10 days | 5/5 normalisation of methane on breath test | No* |
| 15 | Pimentel M et al^15^ | None for the past 3 months | NA | Neomycin | 10 days | No specific comment on methane breath test efficacy | No* |
| 16 | Pimentel M et al ^16^ | NA | NA | No | NA | NA | No |
| 17 | Peled Y et al ^17^ | NA | NA | NA | NA | NA | NA |

IBS: irritable bowel syndrome; SIBO: small intestinal bacterial overgrowth; PPI: proton pump inhibitor; NA: not applicable. *except for cholecystectomy.

**Table S2:** Assessment of risk factors and cut off criteria for methane positive SIBO in IBD patients included in the systematic review and meta-analysis.

| **Study No** | **Author** | **Mode of SIBO diagnosis** | **Dose of substrate** | **Prior antibiotic use** | **Concurrent PPI use** | **Cut off criteria for methane positive SIBO diagnosis** |
| --- | --- | --- | --- | --- | --- | --- |
| 1 | Ricci J et al ^18^ | GBT | 50g | None for the past 1 month | None for the past 1 month | Rise in CH_4_ ≥12 ppm from fasting baseline value on 2 consecutive samplings within 2 hours |
| 2 | Greco A et al ^19^ | GBT | 75g | None for the past 1 month | NA | Rise in CH_4_ ≥12 ppm in at least 3 readings over baseline value |
| 3 | Lee JM et al ^20^ | GBT | 75g | No | No | Rise in CH_4_ ≥ 10 ppm above the baseline |
| 4 | Rana S et al ^21^ | GBT | 80g | NA | No | Rise in CH_4_ ≥12 ppm in 2 consecutive readings within 2 hours |
| 5 | Castiglione F et al ^22^ | LBT | 10g | NA | No | CH_4_ level (≥12–15 ppm) |
| 6 | Peled Y et al^17^ | Measuring breath methane | NA | NA | NA | Breath CH_4_ level was at least 1 ppm above ambient air |
| 7 | Shah A et al^1^ | GBT | 75g | No | 13/81 were on a PPI | Rise in CH_4_ ≥ 10ppm above the baseline |

ppm: parts per million; LBT: lactulose breath test; GBT: glucose breath test; CH_4_: methane; NA: not applicable; OCTT: orocaecal transit time; SIBO: small intestinal bacterial overgrowth; IBD: inflammatory bowel disease.

**Table S3:** Assessment of cut off criteria for diagnosing methane positive SIBO in IBS patients.

| **Study No** | **Author** | **Mode of SIBO diagnosis** | **Dose of substrate** | **Cut off criteria for methane positive SIBO diagnosis** |
| --- | --- | --- | --- | --- |
| 1 | Shah A et al^1^ | GBT | 75g | Rise in CH_4_ ≥ 10 ppm above baseline. |
| 2 | Ghoshal UC et al^2^ | LBT | 10g | CH_4_ ≥ 10 ppm at baseline or rise in CH_4_ ≥10 ppm above baseline. |
| 3 | Ghoshal U et al^3^ | LBT | 10g | CH_4_ ≥ 10 ppm at baseline or rise in CH_4_ ≥10 ppm above baseline. |
| 4 | Vega A et al^4^ | LBT | 10g | CH_4_ > 1 ppm above that of room air at baseline and also showed a CH_4_ value > 3 ppm at some point of the curve. |
| 5 | Lee KN et al ^5^ | LBT | 10g | Rise in CH_4_ > 10 ppm within 90 mins. |
| 6 | Rana S et al^6^ | GBT | 80g | Rise in CH_4_ ≥ 10 ppm above baseline within 120 mins. |
| 7 | Park JS et al^7^ | LBT | NA | CH_4_>10 ppm at baseline or rise in CH_4_ >10 ppm above baseline within 90 mins. |
| 8 | Hwang L et al ^8^ | LBT | 10g | Rise in CH_4_ ≥ 5 ppm above baseline |
| 9 | Parodi A et a ^9^ | GBT | 15g | CH_4_ >10 ppm at baseline or rise in CH_4_ >10 ppm above baseline. |
| 10 | Scarpellini E et al^10^ | LBT | 10g | CH4 >20 ppm within the first 90 minutes. |
| 11 | Bratten J et al^11^ | LBT | 10g | CH_4_ >1 ppm at baseline or at any point during the test. |
| 12 | Majewski M et al^12^ | GBT | 50g | CH_4_ >20 ppm when baseline less than 10 ppm or for any rise in CH_4_ > 12 ppm. |
| 13 | Chatterjee S et al^13^ | LBT | 10g | Presence of methane on breath sample data from at least one point in the test. |
| 14 | Pimentel M et al^14^ | LBT | 10g | Presence of methane in breath samples post lactulose ingestion. |
| 15 | Pimentel M et al^15^ | LBT | 10g | Normal LBT: No rise of H2 or CH4 before 90 mins of lactulose, with a definitive rise never more than 20ppm during 180 mins of test. Rest was classified as abnormal. |
| 16 | Pimentel M et al ^16^ | LBT | 10g | Rise in CH_4_ >20 ppm within 90 mins. |
| 17 | Peled Y et al^17^ | Breath CH_4_ | NA | Breath CH_4_ level was at least 1 ppm above ambient air. |

ppm: parts per million; LBT: lactulose breath test; GBT: glucose breath test; CH_4_: methane; NA: not applicable; SIBO: small intestinal bacterial overgrowth; IBS: Irritable bowel syndrome.

**Table S4:** Studies evaluating the effect of antibiotic treatment in IBS patients with methane positive SIBO.

| **No** | **Author** | **Mode of diagnosis of SIBO** | **Methane positive SIBO patients who underwent treatment,**  **n** | **Antibiotic type** | **Duration of therapy** | **Post treatment symptom improvement**  **n** | **Post treatment Normalization of breath test, n** | **Adverse effects, n** | **Comments** |
| --- | --- | --- | --- | --- | --- | --- | --- | --- | --- |
| 1 | Pimentel et al^14^ | LBT | 5 | Neomycin 500 mg or placebo twice daily | 10 days | Neomycin as compared to placebo was associated with significant improvement in constipation (44.0±12.3% vs 5.0±5.1%, respectively, p<0.05). Symptom improvement was seen in all 5 patients | 5 | NA |  |
| 2 | Vega AB et al^4^ | LBT | 24 | Ispaghula husk three times a day | 4 weeks | Bristol scale score improved, (basal 83±31 vs post treatment 68±3, p<0.05). | In methane producers, treatment resulted in improvement in peak methane (−13 ± 24 ppm; p = 0.014) and methane‐AUC (−817 ± 3100 ppm/min; p = 0.04)., on follow up LBT. | 1 dropped out due to abdominal pain and discomfort, 9 developed excessive flatus and bloating. | In methane producers, treatment resulted in improvement in CTT (−10 ± 35 h; p = 0.029 vs baseline). |
| 3 | Ghoshal UC et al^2^ | LBT | 13 | Rifaximin 400 mg or placebo three times a day | 14 days | Weekly stool frequency (3 [1–9] and 7 [1–14], p = 0.05) and forms improved with rifaximin than placebo. | AUC for methane was lower among patients on rifaximin (6697.5 (1777.5–23,580) vs. 2617.5 (562.5–19,867.5), p = 0.005) than those on placebo (3945 (2415–12,952.5) vs. 3720 (502.5–9210, p = 0.118). | Only one patient experienced transient nausea, while on rifaximin treatment. | CTT normalized in 4/6 (66.7%) on rifaximin but none on placebo (p = 0.02). |

SIBO: small intestinal bacterial overgrowth; BT: breath test; LBT: lactulose breath test; NA: not available; AUC: area under the curve; CTT: colonic transit time.

**Table S5:** Newcastle-Ottawa scale for assessment of quality of case-control studies assessing the prevalence of methane positive SIBO in patients with IBS included in the systematic review and meta-analysis.

|  | Shah A et al^1^ | Ghoshal UC et al^2^ | Vega A et al^4^ | Lee KN et al^5^ | Rana S et al^6^ | Park JS et al^7^ | Parodi A et al^9^ | Scarpellini E et al^10^ | Bratten J et al^11^ | Peled Y et al^17^ |
| --- | --- | --- | --- | --- | --- | --- | --- | --- | --- | --- |
| **SELECTION** |  |  |  |  |  |  |  |  |  |  |
| Is the case definition adequate? | * | * | * | * | * | * | * | * | * | * |
| Representativeness of the cases | * | * | * | * | * | * | * | * | * | * |
| Selection of Controls | - | - | * | * | * | * | * | * | * | * |
| Definition of Controls | * | - | * | * | * | - | - | - | * | * |
| **COMPARIBILITY** |  |  |  |  |  |  |  |  |  |  |
| Study controls for single factor | * | * | * | * | * | * | * | * | * | - |
| Study controls for additional factors | * | - | * | * | * | * | * | * | * | - |
| **EXPOSURE** |  |  |  |  |  |  |  |  |  |  |
| Ascertainment of exposure | * | * | * | * | * | * | * | * | * | - |
| Same method of ascertainment for cases and controls | * | * | * | * | * | * | * | * | * | - |
| Non-Response rate | - | - | - | - | - | - | - | - | - | - |
| **Overall Quality Score**  **(Maximum = 9)** | 7 | 5 | 8 | 8 | 8 | 7 | 7 | 7 | 8 | 4 |

* each asterisk represents if individual criterion within the subsection was fulfilled.

**Table S6:** Joanna Briggs Institute (JBI) Critical Appraisal Tools for quality assessment of prevalence studies and the case group of the case-control studies assessing methane positive SIBO in IBS included in the systematic review and meta-analysis.

|  |  | 1. Was the sample frame appropriate to address the target population? | 2. Were study participants sampled in an appropriate way? | 3. Was the sample size adequate? | 4. Were the study subjects and the setting described in detail? | 5. Was the data analysis conducted with sufficient coverage of the identified sample? | 6. Were valid methods used for the identification of the condition? | 7. Was the condition measured in a standard, reliable way for all participants? | 8. Was there appropriate statistical analysis? | 9. Was the response rate adequate, and if not, was the low response rate managed appropriately? | Risk of bias |
| --- | --- | --- | --- | --- | --- | --- | --- | --- | --- | --- | --- |
| 1 | Shah A et al^1^ | Yes | Yes | Yes | Yes | Yes | Yes | Yes | Yes | N/A | Low |
| 2 | Ghoshal UC et al^2^ | No | Yes | No | Yes | Yes | Yes | Yes | Unclear | N/A | Moderate |
| 3 | Vega A et al^4^ | Yes | Yes | Yes | Yes | Yes | Yes | Yes | Yes | N/A | Low |
| 4 | Lee KN et al^5^ | Yes | Yes | Yes | Yes | Yes | Yes | Yes | Yes | N/A | Low |
| 5 | Rana S et al^6^ | Yes | Yes | Yes | Yes | Yes | Yes | Yes | Yes | N/A | Low |
| 6 | Park JS et al^7^ | Yes | Unclear | Yes | Yes | Yes | Yes | Yes | Yes | N/A | Low |
| 7 | Parodi A et al^9^ | Unclear | Yes | Yes | Yes | Yes | Yes | Yes | Unclear | N/A | Low |
| 8 | Scarpellini E et al^10^ | Unclear | Yes | Yes | Yes | Yes | Yes | Yes | Unclear | N/A | Low |
| 9 | Bratten J et al^11^ | Yes | Yes | Yes | Yes | Yes | Yes | Yes | Yes | N/A | Low |
| 10 | Peled Y et al^17^ | Yes | No | Yes | No | Yes | No | No | No | N/A | High |
| 11 | Ghoshal U et al^3^ | Yes | Yes | No | Yes | No | Yes | Yes | Yes | N/A | Moderate |
| 12 | Hwang L et al ^8^ | Unclear | No | Yes | Yes | Yes | Yes | Yes | Yes | N/A | Moderate |
| 13 | Majewski M et al^12^ | Unclear | Unclear | Yes | Yes | Yes | Yes | Yes | Yes | N/A | Moderate |
| 14 | Chatterjee S at al ^13^ | No | Yes | Yes | Yes | Yes | Yes | No | Unclear | N/A | Moderate |
| 15 | Pimentel M et al ^14^ | No | Yes | No | Yes | Yes | Yes | Yes | Yes | N/A | Moderate |
| 16 | Pimentel M et al ^15^ | No | Yes | Yes | Unclear | Yes | Yes | Yes | Yes | N/A | Moderate |
| 17 | Pimentel M et al ^16^ | Unclear | Yes | Yes | Unclear | Yes | Yes | Yes | Yes | N/A | Moderate |

N/A: not applicable

**Table S7:** Newcastle-Ottawa scale for assessment of quality of case control studies assessing the prevalence of methane positive SIBO in patients with IBD included in the systematic review and meta-analysis.

|  | Ricci J et al ^18^ | Lee JM et al ^20^ | Rana S et al^21^ | Castiglione F et al^22^ | Peled Y et al^17^ | Shah A et al^1^ |
| --- | --- | --- | --- | --- | --- | --- |
| **SELECTION** |  |  |  |  |  |  |
| Is the case definition adequate? | * | * | * | * | * | * |
| Representativeness of the cases | - | * | * | * | * | * |
| Selection of Controls | - | * | * | * | * | - |
| Definition of Controls | - | - | * | * | * | * |
| **COMPARIBILITY** |  |  |  |  |  |  |
| Study controls for single factor | * | * | * | - | - | * |
| Study controls for additional factors | * | * | * | - | - | * |
| **EXPOSURE** |  |  |  |  |  |  |
| Ascertainment of exposure | * | * | * | * | - | * |
| Same method of ascertainment for cases and controls | * | * | * | * | - | * |
| Non-Response rate | - | - | - | - | - | - |
| **Overall Quality Score**  **(Maximum = 9)** | 5 | 7 | 8 | 6 | 4 | 7 |

* each asterisk represents if individual criterion within the subsection was fulfilled.

**Table S8:** Joanna Briggs Institute (JBI) Critical Appraisal Tools for quality assessment of prevalence studies and the case group of the case-control studies assessing methane positive SIBO in IBD included in the systematic review and meta-analysis.

|  |  | 1. Was the sample frame appropriate to address the target population? | 2. Were study participants sampled in an appropriate way? | 3. Was the sample size adequate? | 4. Were the study subjects and the setting described in detail? | 5. Was the data analysis conducted with sufficient coverage of the identified sample? | 6. Were valid methods used for the identification of the condition? | 7. Was the condition measured in a standard, reliable way for all participants? | 8. Was there appropriate statistical analysis? | 9. Was the response rate adequate, and if not, was the low response rate managed appropriately? | Risk of bias |
| --- | --- | --- | --- | --- | --- | --- | --- | --- | --- | --- | --- |
| 1 | Ricci J et al ^18^ | No | No | Yes | Unclear | Yes | Yes | Yes | Yes | N/A | High |
| 2 | Lee JM et al ^20^ | Yes | No | Yes | Yes | Yes | Yes | Yes | Yes | N/A | Low |
| 3 | Rana S et al^21^ | Yes | Yes | Yes | Yes | Yes | Yes | Yes | Yes | N/A | Low |
| 4 | Castiglione F et al^22^ | Yes | Yes | Yes | Yes | Yes | Yes | Yes | Unclear | N/A | Low |
| 5 | Peled Y et al^17^ | Yes | No | Yes | No | No | Unclear | Unclear | No | N/A | High |
| 6 | Shah A et al^1^ | Yes | Yes | Yes | Yes | Yes | Yes | Yes | Yes | N/A | Low |
| 7 | Greco A et al^19^ | Yes | No | Yes | Yes | Yes | Yes | Yes | Yes | N/A | Low |

N/A: not applicable

**Table S9:** Exclusion criteria for studies excluded from the systematic review and meta-analysis.

Full-text articles excluded, with reasons (n = 30),

- Dual publication = 4^5, 8, 11, 23^
- Abstracts only = 3
- Animal study = 2^24, 25^
- Mixed study for FGIDs with no separate data for IBS or IBD patients = 7^26-32^
- Unable to extract clear data which suggested, methane positivity = 14^33-46^

**REFERNCES for supplementary material:**

1. Shah A, Talley NJ, Koloski N, et al. Duodenal bacterial load as determined by quantitative polymerase chain reaction in asymptomatic controls, functional gastrointestinal disorders and inflammatory bowel disease. Aliment Pharmacol Ther 2020;52:155-167.

2. Ghoshal UC, Srivastava D, Misra A. A randomized double-blind placebo-controlled trial showing rifaximin to improve constipation by reducing methane production and accelerating colon transit: A pilot study. Indian J Gastroenterol 2018;37:416-423.

3. Ghoshal U, Shukla R, Srivastava D, et al. Irritable Bowel Syndrome, Particularly the Constipation-Predominant Form, Involves an Increase in Methanobrevibacter smithii, Which Is Associated with Higher Methane Production. Gut Liver 2016;10:932-938.

4. Vega AB, Perelló A, Martos L, et al. Breath methane in functional constipation: response to treatment with Ispaghula husk. Neurogastroenterol Motil 2015;27:945-53.

5. Lee KN, Lee OY, Koh DH, et al. Association between symptoms of irritable bowel syndrome and methane and hydrogen on lactulose breath test. J Korean Med Sci 2013;28:901-7.

6. Rana SV, Sharma S, Kaur J, et al. Comparison of lactulose and glucose breath test for diagnosis of small intestinal bacterial overgrowth in patients with irritable bowel syndrome. Digestion 2012;85:243-7.

7. Park JS, Yu JH, Lim HC, et al. [Usefulness of lactulose breath test for the prediction of small intestinal bacterial overgrowth in irritable bowel syndrome]. Korean J Gastroenterol 2010;56:242-8.

8. Hwang L, Low K, Khoshini R, et al. Evaluating breath methane as a diagnostic test for constipation-predominant IBS. Dig Dis Sci 2010;55:398-403.

9. Parodi A, Dulbecco P, Savarino E, et al. Positive glucose breath testing is more prevalent in patients with IBS-like symptoms compared with controls of similar age and gender distribution. J Clin Gastroenterol 2009;43:962-6.

10. Scarpellini E, Giorgio V, Gabrielli M, et al. Prevalence of small intestinal bacterial overgrowth in children with irritable bowel syndrome: a case-control study. J Pediatr 2009;155:416-20.

11. Bratten JR, Spanier J, Jones MP. Lactulose breath testing does not discriminate patients with irritable bowel syndrome from healthy controls. Am J Gastroenterol 2008;103:958-63.

12. Majewski M, McCallum R. Results of small intestinal bacterial overgrowth testing in irritable bowel syndrome patients: Clinical profiles and effects of antibiotic trial. Advances in medical sciences 2007;52:139-42.

13. Chatterjee S, Park S, Low K, et al. The degree of breath methane production in IBS correlates with the severity of constipation. Am J Gastroenterol 2007;102:837-41.

14. Pimentel M, Chatterjee S, Chow EJ, et al. Neomycin improves constipation-predominant irritable bowel syndrome in a fashion that is dependent on the presence of methane gas: subanalysis of a double-blind randomized controlled study. Dig Dis Sci 2006;51:1297-301.

15. Pimentel M, Chow EJ, Lin HC. Normalization of lactulose breath testing correlates with symptom improvement in irritable bowel syndrome. a double-blind, randomized, placebo-controlled study. Am J Gastroenterol 2003;98:412-9.

16. Pimentel M, Mayer AG, Park S, et al. Methane production during lactulose breath test is associated with gastrointestinal disease presentation. Dig Dis Sci 2003;48:86-92.

17. Peled Y, Weinberg D, Hallak A, et al. Factors affecting methane production in humans. Gastrointestinal diseases and alterations of colonic flora. Dig Dis Sci 1987;32:267-71.

18. Ricci JERJ, Chebli LA, Ribeiro T, et al. Small-Intestinal Bacterial Overgrowth is Associated With Concurrent Intestinal Inflammation But Not With Systemic Inflammation in Crohn's Disease Patients. J Clin Gastroenterol 2018;52:530-536.

19. Greco A, Caviglia GP, Brignolo P, et al. Glucose breath test and Crohn's disease: Diagnosis of small intestinal bacterial overgrowth and evaluation of therapeutic response. Scand J Gastroenterol 2015;50:1376-81.

20. Lee JM, Lee KM, Chung YY, et al. Clinical significance of the glucose breath test in patients with inflammatory bowel disease. J Gastroenterol Hepatol 2015;30:990-4.

21. Rana SV, Sharma S, Malik A, et al. Small intestinal bacterial overgrowth and orocecal transit time in patients of inflammatory bowel disease. Dig Dis Sci 2013;58:2594-8.

22. Castiglione F, Del Vecchio Blanco G, Rispo A, et al. Orocecal transit time and bacterial overgrowth in patients with Crohn's disease. J Clin Gastroenterol 2000;31:63-6.

23. Pimentel M, Mayer AG, Park S, et al. Methane Production During Lactulose Breath Test Is Associated with Gastrointestinal Disease Presentation. Digestive Diseases and Sciences 2003;48:86-92.

24. Kawagoe N, Kijma S, Tanaka H, et al. Alteration of Breath Hydrogen and Methane in Ethanol-Fed Rats. Nihon Arukoru Yakubutsu Igakkai Zasshi 2016;51:403-413.

25. Szűcs S, Bari G, Ugocsai M, et al. Detection of Intestinal Tissue Perfusion by Real-Time Breath Methane Analysis in Rat and Pig Models of Mesenteric Circulatory Distress. Crit Care Med 2019;47:e403-e411.

26. Wilder-Smith CH, Materna A, Wermelinger C, et al. Fructose and lactose intolerance and malabsorption testing: the relationship with symptoms in functional gastrointestinal disorders. Aliment Pharmacol Ther 2013;37:1074-83.

27. Attaluri A, Jackson M, Valestin J, et al. Methanogenic flora is associated with altered colonic transit but not stool characteristics in constipation without IBS. Am J Gastroenterol 2010;105:1407-11.

28. Sung HJ, Paik CN, Chung WC, et al. Small Intestinal Bacterial Overgrowth Diagnosed by Glucose Hydrogen Breath Test in Post-cholecystectomy Patients. J Neurogastroenterol Motil 2015;21:545-51.

29. Wilder-Smith CH, Olesen SS, Materna A, et al. Predictors of response to a low-FODMAP diet in patients with functional gastrointestinal disorders and lactose or fructose intolerance. Aliment Pharmacol Ther 2017;45:1094-1106.

30. Lee KM, Paik CN, Chung WC, et al. Breath methane positivity is more common and higher in patients with objectively proven delayed transit constipation. Eur J Gastroenterol Hepatol 2013;25:726-32.

31. Martínez-Azcona O, Moreno-Álvarez A, Seoane-Pillado T, et al. Fructose malabsorption in asymptomatic children and in patients with functional chronic abdominal pain: a prospective comparative study. Eur J Pediatr 2019;178:1395-1403.

32. Waingankar K, Lai C, Punwani V, et al. Dietary exclusion of fructose and lactose after positive breath tests improved rapid-transit constipation in children. JGH Open 2018;2:262-269.

33. Wolf PG, Parthasarathy G, Chen J, et al. Assessing the colonic microbiome, hydrogenogenic and hydrogenotrophic genes, transit and breath methane in constipation. Neurogastroenterology and motility : the official journal of the European Gastrointestinal Motility Society 2017;29:1-9.

34. Di Stefano M, Mengoli C, Bergonzi M, et al. Breath Methane Excretion Is not An Accurate Marker of Colonic Methane Production in Irritable Bowel Syndrome. Am J Gastroenterol 2015;110:891-8.

35. Kim G, Deepinder F, Morales W, et al. Methanobrevibacter smithii is the predominant methanogen in patients with constipation-predominant IBS and methane on breath. Dig Dis Sci 2012;57:3213-8.

36. Kasir R, Zakko S, Zakko P, et al. Predicting a Response to Antibiotics in Patients with the Irritable Bowel Syndrome. Dig Dis Sci 2016;61:846-51.

37. Ding XW, Liu YX, Fang XC, et al. The relationship between small intestinal bacterial overgrowth and irritable bowel syndrome. Eur Rev Med Pharmacol Sci 2017;21:5191-5196.

38. Rana SV, Sinha SK, Sharma S, et al. Effect of predominant methanogenic flora on outcome of lactose hydrogen breath test in controls and irritable bowel syndrome patients of north India. Dig Dis Sci 2009;54:1550-4.

39. Vernia P, Camillo MD, Marinaro V, et al. Effect of predominant methanogenic flora on the outcome of lactose breath test in irritable bowel syndrome patients. Eur J Clin Nutr 2003;57:1116-9.

40. Almazar AE, Chang JY, Larson JJ, et al. Comparison of Lactase Variant MCM6 -13910 C>T Testing and Self-report of Dairy Sensitivity in Patients With Irritable Bowel Syndrome. J Clin Gastroenterol 2019;53:e227-e231.

41. DuPont AW, Jiang ZD, Harold SA, et al. Motility abnormalities in irritable bowel syndrome. Digestion 2014;89:119-23.

42. Makhani M, Yang J, Mirocha J, et al. Factor analysis demonstrates a symptom cluster related to methane and non-methane production in irritable bowel syndrome. J Clin Gastroenterol 2011;45:40-4.

43. Reddymasu SC, Sostarich S, McCallum RW. Small intestinal bacterial overgrowth in irritable bowel syndrome: are there any predictors? BMC Gastroenterol 2010;10:23.

44. Furnari M, Savarino E, Bruzzone L, et al. Reassessment of the Role of Methane Production between Irritable Bowel Syndrome and Functional Constipation. Journal of gastrointestinal and liver diseases : JGLD 2012;21:157-63.

45. Sachdeva S, Rawat AK, Reddy RS, et al. Small intestinal bacterial overgrowth (SIBO) in irritable bowel syndrome: frequency and predictors. J Gastroenterol Hepatol 2011;26 Suppl 3:135-8.

46. Suri J, Kataria R, Malik Z, et al. Elevated methane levels in small intestinal bacterial overgrowth suggests delayed small bowel and colonic transit. Medicine (Baltimore) 2018;97:e10554.
